# Physical Activity and Health Promotion in Esports and Gaming–Discussing Unique Opportunities for an Unprecedented Cultural Phenomenon

**DOI:** 10.3389/fspor.2021.693700

**Published:** 2021-09-16

**Authors:** Sascha Ketelhut, Anna Lisa Martin-Niedecken, Patrick Zimmermann, Claudio R. Nigg

**Affiliations:** ^1^Department of Health Science, Institute of Sport Science, University of Bern, Bern, Switzerland; ^2^Subject Area in Game Design, Department of Design, Zurich University of the Arts, Zurich, Switzerland

**Keywords:** gamers, health promotion, physical activity, performance enhancement, exergames, digital natives, esports

## Abstract

Due to long periods of sedentary behavior, and unhealthy diets gamers and esports players are at risk for numerous chronic diseases and all-cause mortality. Health research has started addressing the public health implications of the esports phenomenon, drawing a bleak picture of this megatrend. However, instead of just highlighting adverse public health implications of this trend, it is recommended to become involved in this phenomenon and positively influence it. Esports has an enormous potential for physical activity and health-promoting efforts, provides a context for broadly disseminating interventions, and offers new ways of gaining access to an often-neglected population. This paper presents: the potential of the esports phenomenon to promote physical activity, health, and well-being in gamers and esports players; the strategic and preventive solutions to ameliorate esports possible adverse health impacts; and the utilization of esports technology (streams, media platforms, exergames, etc.) as an innovative health promotion tool, especially reaching gamers and esports players with attractive and interactive interventions. This is to encourage systematic scientific research so that evidence-based guidelines and intervention strategies involving regular physical activity, healthy diet, and sleep hygiene for esports will be developed. The goal is to promote public health approaches that move toward a better integration of esports and gaming.

## Introduction

Physical inactivity, sedentary behavior (SB), and unhealthy diets are major causes of non-communicable diseases and premature death (Lee et al., 2012; Wilmot et al., 2012). As esports—serious competitive video gaming—has been associated with these unhealthy behaviors, esports developments are often regarded as questionable, especially from a public health perspective (Borggrefe, 2019; Yin et al., 2020). Researchers started addressing the public health implications of the esports phenomenon, drawing a bleak picture of this megatrend (Wattanapisit et al., 2020). Commonly identified adverse health outcomes related to esports and gaming include increased stress (Smith et al., 2019), sleep disturbances (Peracchia and Curcio, 2018), obesity (DiFrancisco-Donoghue et al., 2020), and behavioral problems (Yin et al., 2020). Relatedly, gaming time is linked to sedentary behavior (Rudolf et al., 2020) which also is associated with a higher risk for non-communicable diseases (Bailey et al., 2019). Additionally, esport participants are at risk for problematic Internet use (Argyriou et al., 2017). However, instead of just illustrating the adverse side effects and public health implications, it seems more advisable to understand this phenomenon and leverage the opportunities it offers. Just by its sheer dimension, esports has enormous potential as a setting to promote physical activity (PA) and health. It is important to improve our understanding of how to promote PA, health, and well-being and identify strategic and preventive solutions to address possible adverse health impacts in the esports community.

This paper presents some unique opportunities the esports phenomenon offers to influence PA and health in the esports community. It further tries to encourage systematic scientific research to develop evidence-based guidelines and intervention strategies for health promotion. This is intended to promote public health approaches that move toward a better integration of an ever-growing population of esports players, gamers, and audience.

## The Esports and Gaming Community

Esports players and gamers are stereotypically portrayed as young, male, socially inept, isolated “couch potatoes” (Williams, 2003). However, modern video games are not isolating activities that come at the expense of social interaction. Especially multiplayer online games are vibrant community sites (Steinkuehler and Williams, 2006). Research on gamer populations suggests that modern gamers are not isolated teenage males (Yee, 2006; Hedlund, 2021), and that video games are no longer child's play. The average gamer is older than 30 years (Hedlund, 2021). Millennials make up a large portion of the gaming community. Besides playing games with the primary intention of pure enjoyment and entertainment, people from all continents and all walks of life practice or watch esports. Also, the gender composition is changing to an increasing number of highly competitive female esports players (McLean and Griffiths, 2013; Hedlund, 2021).

An esports viewer is usually a video gamer that often wants to learn something about a particular game and the esports audience is mainly made up of millennials (Hamilton et al., 2014; Nilsen, 2019; Xiao, 2020). About 71% of millennials watch gaming content, consuming almost 6 h of gaming content each week (Nilsen, 2019). It is evident that esports is no longer a niche but has arrived in society's mainstream. By reaching the esport community, we potentially gain access to a significant and continuously growing part of our society.

## Physical Activity in the Esports Community

PA is considered a vital component of a healthy lifestyle as seen in guidelines of different professional societies (Piercy et al., 2018). Adults should do at least 150–300 min of moderate-intensity aerobic PA; or at least 75–150 min of vigorous-intensity aerobic PA; or an equivalent combination of moderate- and vigorous-intensity activity throughout the week (World Health Organisation, 2020). Despite the benefits of regular PA (Lee et al., 2012), around one-fourth of the world's population do not meet the WHO guidelines (Guthold et al., 2018). Technological advances, societal influences, changes in transportation, and environment have shaped our daily life, decreasing PA, and increasing SB. A growing body of evidence has linked SB to several adverse health outcomes, including obesity, metabolic syndrome, and cardiovascular disease (Katzmarzyk et al., 2009; Marker et al., 2019). Media use is often implicated for causing physical inactivity and increasing SB (Duncan et al., 2012; Hingle and Kunkel, 2012).

Esports players are often referred to as sedentary athletes as gaming requires prolonged sedentary activity. Esports players of different performance levels accumulate more than 3.5 h of gaming per day (Rudolf et al., 2020), while competitive elite esport players reach ~5.3 h per day (Kari and Karhulahti, 2016). Leading up to competitions, collegiate esports athletes reported to practice between 5.5 and 10 h daily (DiFrancisco-Donoghue et al., 2019).

Current data on esports players' PA show inconsistent results; according to an international online survey conducted by Trotter et al. (2020), 80.3% of esports players do not meet the WHO's PA recommendations. Thus, the prevalence of physical inactivity in esports players seems higher than in the general population (Guthold et al., 2018). Contrary, a study by Rudolf et al. (2020) determined that two-thirds of the esport players in Germany achieve the WHO's PA recommendations. This is in line with DiFrancisco-Donoghue et al. (2019), who reported that only about 40% of collegiate esport players from the USA and Canada did not participate in regular PA.

Gaming is typically associated with a higher body mass index (BMI) and, as the length of video gaming sessions is positively associated with BMI (Ballard et al., 2009), esports players may display a higher BMI compared to the general population. According to Trotter et al. (2020), esports players were more likely to be of normal weight, however, they were also more likely to be morbidly obese than the global population. In the study by Rudolf et al. (2020), 51% of the esports players were classified as normal weight. DiFrancisco-Donoghue et al. (2020) found no difference between collegiate esports players and age- matched controls in BMI. Esports players, however, had significantly higher total body fat percentage and significantly lower total lean mass.

Currently, it is difficult to draw conclusions on the PA and health status of esports players. Most available data is cross-sectional and only based on online surveys, not allowing to establish causality. To date, large-scale accelerometer-based and longitudinal cohort studies are missing. Furthermore, many studies are methodologically flawed as they do not control for potential confounding factors like gender, age, game type, and performance level. Pereira et al. (2019) show that increasing competitiveness affects PA engagement in esports players. Players with a higher in-game rank are more likely to be physically active (Trotter et al., 2020). In a study on high-performance esports athletes, PA levels were three times higher than the WHO guidelines (Kari and Karhulahti, 2016).

Furthermore, studies did not compare the gamers to age matched controls when classifying their sample. A recent study compared accelerometer count in esports players to non-esports players, showed that collegiate esports players were significantly less active (DiFrancisco-Donoghue et al., 2020). Also Rudolf et al. (2020) stated PA status in their study was lower in esports players vs. age matched controls. Due to these methodological deficiencies, the health and PA status of esports players and gamers may be worse than the literature portrays. Regardless, the sedentary nature of gaming puts gamers at a higher health risk. To compensate for this higher risk all efforts should be undertaken to decrease SB and increase PA levels in this population. The esports ecosystem provides promising opportunities.

## Opportunities in Esports

### Digital Technologies

In the context of PA and health promotion, technology is often perceived as a double-edged sword. On the one hand, it contributes to the increase in SB by expanding screen based entertainment services (video games, applications, television shows), and automating operations increasing occupational sitting time (Clark and Sugiyama, 2015). On the other hand, the widespread use of fitness trackers, smartphone applications, and wearable technologies offers innovative solutions (nudging, ambulatory assessment, tracking) to promote healthy behavior changes, motivate the user to increase PA, and foster consumer empowerment (Spanakis et al., 2016; Wortley et al., 2017). Technology potentially overcomes many barriers associated with traditional face-to-face PA programs. Technology-based interventions are constantly accessible, cost-effective, and easy to use (Bacigalupo et al., 2013; Thomas and Bond, 2014). Furthermore, they can remotely monitor patients, thus providing data on factors that influence health behaviors and provide tailored feedback at the appropriate time and place. Technology has the potential to translate evidence-based techniques of behavioral interventions into formats that can be broadly disseminated and individually tailored.

Smartphone applications, wearables and online platforms have been widely used to help track and comprehend health goals, trigger social support, and social comparison (Lewis et al., 2017). Especially so-called digital natives (Palfrey and Gasser, 2008) seem to present the optimal target group. According to Rennis et al. (2015), they are the highest users of the internet and new technologies for communication, recreation, and information gathering. Furthermore, they are more technically advanced than digital immigrants, create more online content, and get more involved in social media (Williams et al., 2012). As most gamers can be referred to as digital natives with a high use of technological devices, this venue may be an ideal platform for the administration of PA and health-promoting interventions.

Especially interventions using mobile phones and wearables, are efficacious for increasing PA and decreasing SB in different target groups (Lewis et al., 2017; Aldenaini et al., 2020). According to a recent review 79% of mobile phone-based studies reported successful outcomes and show to be especially effective in young adults (Aldenaini et al., 2020). Recently mobile technology has been growing dramatically as gaming devices, and mobile esports games have gained popularity (Newzoo, 2019). Thus, utilizing mobile phone-based approaches to promote PA may be especially promising in the esports community.

**Figure 1:**
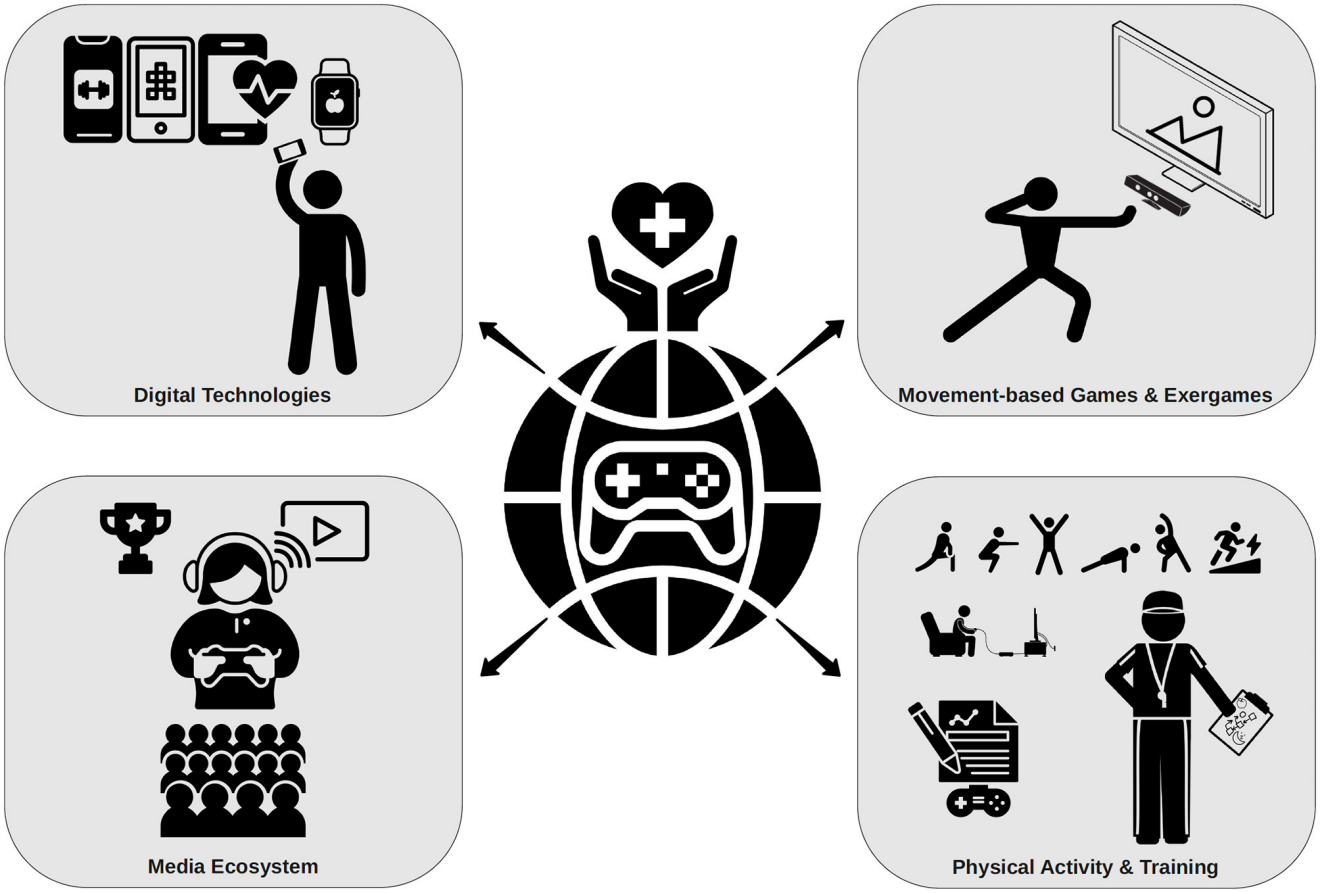
Overview of potential health promotion strategies in esports.

Health promotion specialists should start tailoring digital technology-based intervention strategies for gamers and esports players (Figure 1). It is expected that typical game-like features, social support strategies, social competition, and gamification are promising approaches to address physical inactivity and unhealthy lifestyles in the esports society.

### Movement-Based Games and Exergames

In general, humans, whom Huizinga (Huizinga, 1938) also referred to as “Homo Ludens”—because of their culturally anchored, natural play instinct—are highly motivated through games. Therefore, paradoxically, one promising avenue to encourage esports players to be more physically active could be with video games. Games are defined as intrinsically motivated activities (Salen et al., 2004), which—besides providing entertaining experiences and easy access—allow for incorporating overarching purposes (i.e., learning and training skills). Games with such a characterizing goal are labeled as serious games (Caserman et al., 2020).

Exergames (Oh and Yang, 2010) belong to this genre and have often been described as attractive and effective training tools (Martin-Niedecken et al., 2019; Marshall and Linehan, 2020). Exergames combine physical and cognitive activities with immersive game experiences. Here, the player is physically active throughout the game, must respond to a wide variety of cognitive as well as multi-sensory stimuli, and perform movements to successfully control the game. Depending on the exergame design (movement concept, controller technology, platform, audio-visual design, game mechanics, etc.), the training foci can guide in specific directions. In-game assessments could also enable exergame-based individual training planning and control (Martin-Niedecken et al., 2021).

Due to their playful, motivating, inclusive, and easily accessible nature, exergames could offer a somewhat familiar and thus attractive training approach for esports players and gamers. According to the literature, exergames can increase PA adherence, as players find virtual versions of traditional exercises more enjoyable (Farrow et al., 2019). Furthermore, exergames could help the physically inactive to gain confidence in their movement skills, when practicing a range of motor skills by playing in a safe environment. By simultaneously stimulating cognitive, physical, and mental skills, they could potentially provide beneficial training effects, and positively affect esports player's health and performance (Herold et al., 2018; Martin-Niedecken and Schättin, 2020).

Moreover, esports genres incorporating motion-enhancing, mediating cross-spatial immersive technologies such as augmented reality (AR), mixed reality (MR), or virtual reality (VR) break up the familiar setting in front of the screen. Besides enhancing the involvement of the whole body in the gameplay, they encourage players to walk around, and socialize while practicing their game. AR specifically is a promising concept in that it allows for geographic tailoring to an individual's environment (An and Nigg, 2017). MR- and VR-based esports genres such as Beat Saber, Eco Arena, or the ExerCube (Martin-Niedecken and Schättin, 2020; Martin-Niedecken et al., 2020) even require (intense) PA to successfully play the game. These games and their respective leagues further represent a new era of competition in physical esports (Martin-Niedecken and Schättin, 2020).

Therefore, esports coaches should consider including such beneficial, playful training technologies into existing workout routines of esports players, potentially increasing both PA and in game performance (Figure 1).

### Media Ecosystem

Today, it is possible to disseminate information and opinions via social media worldwide in real-time for free. The potential to increase visibility rapidly and inexpensively is increasingly utilized for the delivery of public health campaigns (Maher et al., 2014). Awareness, recognition, reach, and engagement are known precursors for the effectiveness of health campaigns (Lelutiu-Weinberger et al., 2015; Hair et al., 2017). Thus, social media platforms, can enhance the effectiveness of health promotion interventions by providing access to at-risk or “hidden” audiences and increasing engagement (Kostygina et al., 2020). Especially for young people, social media creates a relevant environment where they are most likely to access and engage with health information (Ahola Kohut et al., 2018). In recent years, so-called “Influencers” have been identified as promising public health agents who disseminate and encourage health behaviors (Hoffman and Tan, 2015).

Esports represents a holistic media ecosystem, which makes it the perfect setting for sharable content and influencer marketing as strategies for health message dissemination. Even though there are live events, the main interaction occurs via online game streaming or gaming platforms. Millions of users watch other gamers play tournaments and in professional leagues through live streams, resulting in an intensive connection to gaming celebrities.

Thus, the esports ecosystem can be used to easily reach an exponentially growing viewership that already surpassed the viewership of most traditional sports (Kane and Spradley, 2017). The digital nature of esports allows more interaction and engagement of the fans than most traditional sports. Fans can follow their idols not only during official tournaments but also during online streaming sessions. Within these streaming sessions, personal interaction between fans and professional esports players is easily facilitated (Cranmer et al., 2021).

Also more casual players stream esports games on their personal channels drawing substantial attention. These influencers have the chance to shape the attitudes and behaviors of millions of followers through their channels. The live-broadcasting nature of the streaming platforms offers a unique relationship between the media creator and media consumer, thus facilitating communication (Sjöblom and Hamari, 2017). Due to the ubiquity of mobile devices, these influencers are becoming constant companions of their followers, making them increasingly pervasive and powerful.

Health professionals should think about harnessing cultural elements endemic to the esports scene to increase the appeal of health campaigns (Figure 1). The highly interactive, immersive, and engaging online environment presents an opportunity to easily reach an ever-growing target group irrespective of cultural background.

### Physical Activity to Enhance Esports Performance

Esports is becoming more professional, with revenues surpassing those of most traditional sports and salaries reaching those of superstar athletes (Roundhill, 2020; Reyes, 2021). These developments will inevitably lead to more people to engage in competitive esports. Players already dedicate much time to develop expertise and to realize their potential. However, the time that can be dedicated to gameplay is limited. Similar to traditional sports, esports players and coaches realize that training by just playing the game at some point will not be enough. It is more effective to practice specific skills in non-gaming environments and look for new opportunities that increase training efficiency.

Research shows that physical exercise can positively modulate the anatomy, physiology, and brain function and hence improve cognitive performance (Hillman et al., 2008; Chang et al., 2012). According to Toth et al. (2020) attention, memory, information processing and task-switching abilities are abilities specifically linked to success in gaming. These executive control processes, relevant for the performance in most esports games (Bediou et al., 2018), are affected by exercise (Kramer and Colcombe, 2018). Especially aerobic exercise seems effective to enhance attentional ability (Toth et al., 2020).

Furthermore, exercise has a mood-enhancing effect and can help reduce anxiety and stress (Callaghan, 2004), which may also positively influence gaming performance. Short bouts of intense exercise before video game playing enhanced game performance during a game of League of Legends (De Las Heras et al., 2020). It is to expect that other healthy lifestyle choices may also positively affect skills acquisition and performance in esports.

Like traditional athletes, esports players can also suffer career-ending injuries (Emara et al., 2020). According to DiFrancisco-Donoghue et al. (2019), esports players are susceptible to chronic overuse injuries as gaming on a competitive level requires players to play for many hours a day. This comprises prolonged sitting in the same position, unphysiological posture, and repetitive movements of small muscle groups.

Esports players may also face mental hazards; with the professionalization of esports and increased social and media impact, the pressure to perform increases. This could predispose esports players to experience anxiety, burnout, or other mental problems, resulting in impaired performance and drop-out.

As regular physical exercise has physiological and mental health benefits, this could present a promising approach for injury prevention and improving cognitive well-being in gamers. Comprehensive physical training interventions may help optimize skills, maximize performance, and as a positive side effect, improve health.

The ambition of esports players, coaches, and sponsors to enhance performance and prevent drop-out may present a gateway for health promotion campaigns and convince players as well as officials to integrate physical exercise into the training routine (Figure 1).

## Future Directions

To reformulate the esports phenomena and compensate for its sedentary nature, efforts need to be undertaken to make esports a powerful player in the health promotion arena. Three areas present unique opportunities: understand to communicate through this group worldwide, address the esports ecosystem to develop tailored intervention strategies, and understand the performance-enhancing effects of increasing PA and decreasing SB.

The reach and influence of esports is now truly global. This is not only true for the players but even more so for the audience. Thus, esports participants interact and communicate on a global scale—understanding this influence and harnessing this to disseminate health promotion efforts can result in an unprecedented reach integrating cultures that share a common passion.

Esports, in many respects, presents a unique environment. Research should start to identify the determinants that either facilitate or impede healthy behaviors resulting from game playing (Yin et al., 2020). Fully understanding this environment which includes focused attention, emotions, interactive gaming technology, competition, cooperation and self-improvement, personalized online environment, among other aspects, makes this a unique platform to develop health promotion efforts from a bio-psycho-social and online environment framework which can incorporate real-time adaptive interventions. As such, theory-based approaches are recommended to improve our understanding of the mechanisms, guide intervention development, and increase effectiveness.

The other area of research involves investigating the effects of increased PA and reduced SB on esports performance. Understanding the esport specific performance effects of PA will provide the rationale for incorporating health into esports training. Developing healthier esports athletes may also result in a role-modeling effect. As such esports trainers could also be conceptualized as health effect multipliers while improving performance.

Considering the potential public health impact (Reach X Efficacy X Retention) (Marcus et al., 2000), the esports phenomenon is potentially the most impactful health investment currently.

## Data Availability Statement

The original contributions presented in the study are included in the article/supplementary material, further inquiries can be directed to the corresponding author.

## Author Contributions

All authors listed contributed to the theoretical development, the drafting, editing of the manuscript, and approved it for publication.

## Conflict of Interest

The authors declare that the research was conducted in the absence of any commercial or financial relationships that could be construed as a potential conflict of interest.

## Publisher's Note

All claims expressed in this article are solely those of the authors and do not necessarily represent those of their affiliated organizations, or those of the publisher, the editors and the reviewers. Any product that may be evaluated in this article, or claim that may be made by its manufacturer, is not guaranteed or endorsed by the publisher.
